# A Case of Direct-Acting Oral Anticoagulant-Induced Intramural Colon Hematoma Successfully Treated by Laparoscopic Surgery

**DOI:** 10.7759/cureus.58513

**Published:** 2024-04-18

**Authors:** Daisuke Tomita, Shigeo Toda, Ryo Miyazaki, Shuichiro Matoba, Hiroya Kuroyanagi

**Affiliations:** 1 Department of Gastroenterological Surgery, Toranomon Hospital, Tokyo, JPN

**Keywords:** laparoscopic surgery, bowel obstruction, direct-acting oral anticoagulant, gastric intramural hematoma, case report

## Abstract

Intramural intestinal hematoma is a rare disease, one of the triggering factors of which is the use of anticoagulants. In previous reports, most patients were on treatment with warfarin. Herein, we report a case of direct-acting oral anticoagulant (DOAC)-induced intramural hematoma of the ascending colon in a patient refractory to conservative treatment and required laparoscopic right hemicolectomy.

An 80-year-old male patient with a history of atrial fibrillation and cerebral infarction, on treatment with apixaban, was brought to our hospital with the chief complaints of abdominal pain, vomiting, and melena. Imaging revealed the cause of symptoms to be intestinal obstruction caused by a mass lesion on the wall of the ascending colon. We initially opted for conservative treatment with discontinuation of apixaban and insertion of an ileus tube. Intestinal dilatation findings showed improvement; however, subsequent imaging examinations did not reveal the shrinkage of a lesion in the ascending colon. If the mass was not removed, recurrence of bowel obstruction symptoms was expected, so we decided to perform surgical intervention. A laparoscopic right hemicolectomy was performed, and an intramural hematoma of the ascending colon was diagnosed based on the excised specimen. He needed a blood transfusion for anemia but was discharged on postoperative day 14 with no other complications.

DOACs are now widely used in patients with atrial fibrillation, and the risk of bleeding as a side effect is extremely low compared to conventional anticoagulants, including warfarin. However, when abdominal pain occurs, as in the present case, an intramural hematoma should be considered in the differential diagnosis. There is no established treatment plan for intestinal intramural hematoma. Although conservative treatment is effective in some cases, it is difficult to evaluate the risk of bleeding associated with DOACs using coagulation tests. Even if conservative treatment is selected, it is essential to determine surgical resection, if necessary, based on the clinical course and imaging and blood test findings.

## Introduction

Intramural hematomas can occur anywhere from the esophagus to the rectum, but the duodenum is the most common site, and intramural hematomas of the colon are rarely reported [1,2]. This condition is generally associated with blunt trauma or complications of anticoagulant therapy as well as other factors such as bleeding diathesis, leukemia, lymphoma, chemotherapy, vasculitis, and collagen diseases [1,3]. A retrospective epidemiologic survey reported that intramural hematomas of the small intestine occur in one out of every 2,500 patients receiving anticoagulation therapy [4]. The likelihood of an intramural colonic hematoma is expected to be even lower. Many patients with intestinal intramural hematomas associated with anticoagulant use warfarin [5]. Since their development in 2011, direct-acting oral anticoagulants (DOACs) have been commonly used to prevent thrombosis in patients with atrial fibrillation and those undergoing hip or knee replacement surgery. Bleeding is a major side effect associated with DOACs, but the risk of bleeding with DOACs is extremely low compared with warfarin [6]. Here, we report a case of intramural hematoma of the ascending colon induced by apixaban, a DOAC, with the patient showing symptoms of intestinal obstruction. Intramural colon hematoma caused by DOAC administration is extremely rare; therefore, we present this case along with a literature review.

## Case presentation

**Figure 1:**
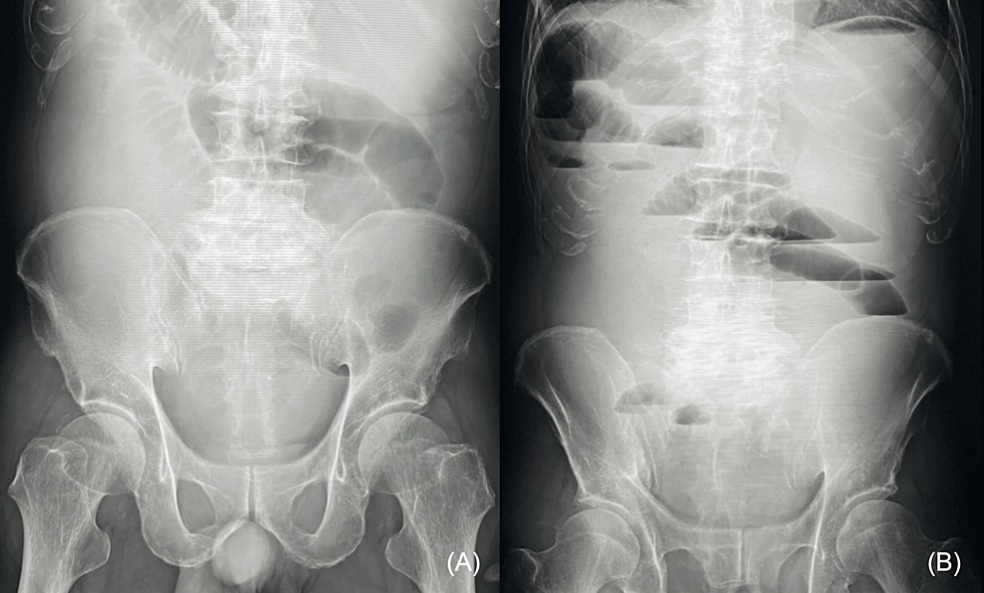
Abdominal radiographs A supine radiograph indicates a dilated small bowel with air-fluid levels (A). An upright radiograph shows multiple air-fluid levels (B).

An 80-year-old Japanese man with a history of hypertension, spinal canal stenosis, chronic gastritis (post-Helicobacter pylori eradication), gastroesophageal reflux disease, cerebral infarction, and atrial fibrillation with a CHA2DS2-VASc score of 7 was presented to the emergency department with the chief complaints of abdominal pain, vomiting, and melena that lasted for three days. The patient was on treatment with olmesartan, pregabalin, limaprost (limaprost alfadex), esomeprazole magnesium hydrate, and apixaban. He didn't have a family history of significant diseases. The vomitus contained blackish blood. The vital signs were stable, and the Glasgow Coma score was 15/15. During the physical examination, there was a slight distension of the abdomen with mild tenderness noted throughout. No guarding or rebound tenderness was detected. The patient had no history of bleeding or abdominal trauma. Upper and lower gastrointestinal endoscopies were performed within one month of the onset of abdominal pain, and no abnormal findings were found at that time. A complete blood cell count showed a leukocyte count of 6,000/μL, mild anemia with a hemoglobin level of 11.6 g/dL, and a platelet count of 12.4 × 10^4/μL. Serum biochemistry studies revealed a slight inflammatory reaction, with a C-reactive protein level of 2.61 mg/dL, creatinine level of 0.72 mg/dL, and slightly elevated blood urea nitrogen (25 mg/dL) and total bilirubin (3.7 mg/dL) levels. Coagulation tests showed an activated partial thromboplastin time of 34.2 seconds and a prothrombin time-international normalized ratio (PT-INR) of 1.39. The level of the tumor markers (carcinoembryonic antigen and carbohydrate antigen 19-9) was within normal limits. Abdominal supine radiography revealed a dilated small bowel with air-fluid accumulation (Figure 1A). Multiple air-fluid levels were observed on upright radiography (Figure 1B).

**Figure 2:**
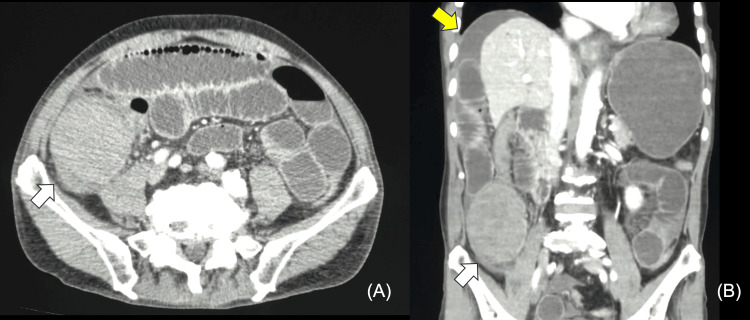
Computed tomography images on the day of admission A hyperdense mass measuring 9.5 cm × 6 cm × 5 cm long is observed in the ascending colon (white arrow). The intestinal tract on its oral side is dilated. Mild ascites is observed on the surface of the liver (yellow arrow). (A) Axial view. (B) Coronal view.

Contrast-enhanced computed tomography (CECT) revealed wall thickening with a narrowing of the lumen in the ascending colon. A hyperdense mass with heterogeneous enhancement measuring 9.5 cm x 6 cm x 5 cm was also observed. The part of the intestine closer to the mass was dilated at the air-fluid level. Additionally, moderate peritoneal effusion was noted on the liver surface (Figures 2A-2B). 

**Figure 3:**
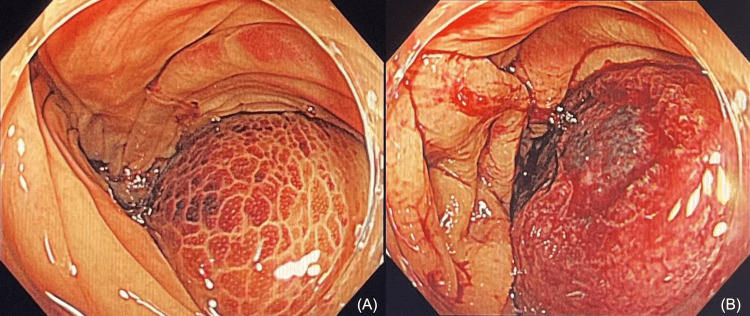
Colonoscopy findings (A) A dark-reddish submucosal tumor-like lesion in the ascending colon. (B) Microvessels are exposed on the surface of the tumor, and minor bleeding is observed.

Upon admission, the patient discontinued all medications and underwent an endoscopy to insert a long tube to relieve the bowel obstruction. The abdominal symptoms improved within a few days after the procedure. On the fourth day after admission, a colonoscopy revealed a dark-reddish submucosal tumor-like lesion with congestion and redness in the ascending colon (Figure 3A). Microvessels were exposed on the surface of the lesion and minor bleeding was observed in the same area (Figure 3B).

**Figure 4:**
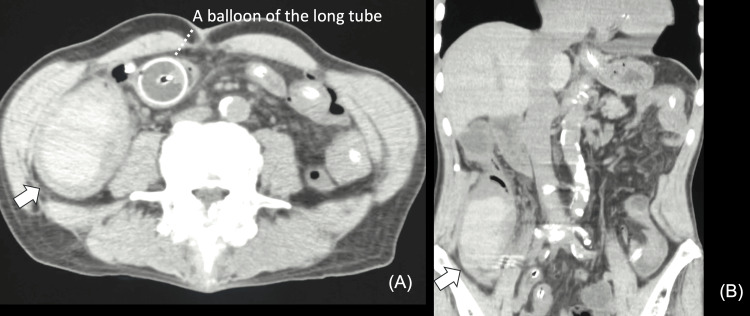
Computed tomography images on the eighth day of admission The tumor is not shrinking (arrow). The intestinal tract is decompressed and ascites is reduced. (A) Axial view. (B) Coronal view.

Based on colonoscopy findings, an intramural hematoma of the ascending colon was suspected, and conservative treatment was initially planned. However, a non-contrast CT scan on the eighth day of hospitalization showed no shrinkage in tumor size, and the possibility of malignant disease could not be ruled out (Figures 4A-4B). Therefore, surgical intervention was selected as the diagnostic treatment.

**Figure 5:**
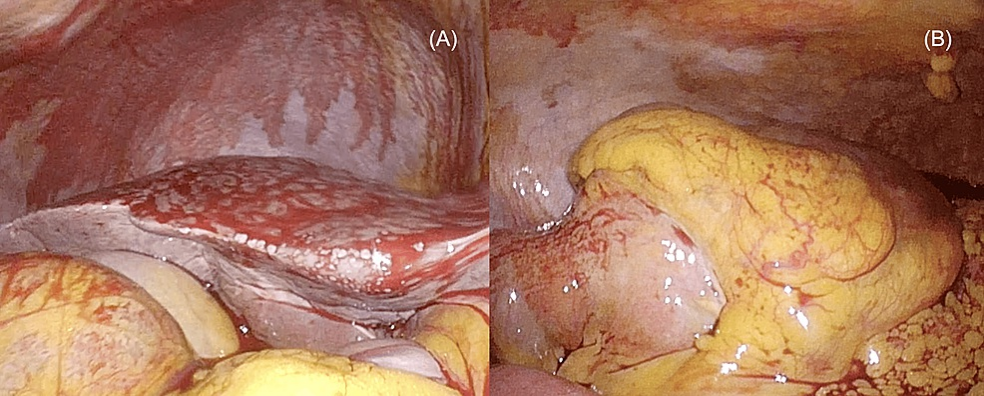
Intraoperative findings (A) Bloody ascites around the liver. (B) Tumor in the ascending colon.

Laparoscopic right hemicolectomy was performed on day 12 of hospitalization. Intraoperative findings revealed bloody ascites around the liver and a tumor in the ascending colon (Figures 5A-5B). The ascending colon was mobilized from the retroperitoneum, and the ileocecal vein and artery were divided at the root. The remaining ileum and transverse colon were anastomosed using the functional end-to-end technique. The surgery was safely completed without intraoperative complications, such as major bleeding. The operation lasted 195 minutes, and the blood loss was approximately 100 mL.

**Figure 6:**
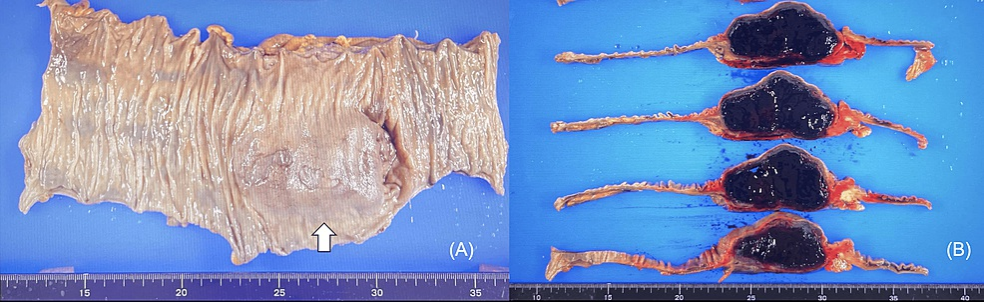
The specimen (A) Submucosal tumor in the ascending colon (arrow). (B) Hematoma formation beneath the submucosa.

The specimen revealed a 9 cm large submucosal tumor in the ascending colon (Figure 6A). The cut surface showed that the hematoma had formed mainly in the intrinsic muscular layer under the submucosa (Figure 6B). No malignancies were detected.

The patient restarted oral intake on the second postoperative day. Apixaban was resumed on the fourth day but had to be discontinued on the sixth day because of the appearance of melena. At that time, the patient's hemoglobin levels were low (7.2 g/dL), and four units of red blood cell concentrate were transfused. Apixaban was resumed on the 11th post-operative day when the hemoglobin levels improved (11.4 g/dL). There were no further complications, and the patient was discharged on the 14th day after the surgery. Forty months after the surgery, there has been no recurrence of the intramural hematoma.

## Discussion

Non-traumatic hematomas of the intestinal wall are mainly caused by anticoagulation therapy, especially with warfarin [5]. Warfarin is a drug that needs dose adjustments based on PT-INR, and altering the dosage may cause a bleeding tendency. Interactions between concomitant medications have been suggested as the cause of an excessively anticoagulated state during warfarin administration [7]. However, DOACs do not affect the action of vitamin K, and drug interactions are rare. Therefore, the possibility of bleeding tendency is significantly lower with DOACs compared to warfarin. A recent study comparing the effectiveness and safety of DOACs with warfarin reported that the combined risk of ischemic stroke, systemic embolism, and death, as well as adverse events such as intracranial hemorrhage, gastrointestinal bleeding, and other minor bleeding events, were lower for DOACs than for warfarin [6,8].

Only one case of DOAC-induced intestinal wall hematoma has been reported so far [9], in which a 73-year-old woman who had received apixaban for atrial fibrillation was diagnosed with small bowel intussusception with obstruction upon imaging studies. The patient underwent exploratory laparotomy and small bowel resection. Histopathological examination revealed that the intussusception was caused by an intramural hematoma.

Our patient was on treatment with limaprost alfadex, a prostaglandin E1 derivative that inhibits platelet aggregation, in addition to apixaban, which increased the risk of bleeding events compared with the previous case. Although DOACs have a relatively low risk of bleeding, they are increasingly used with other drugs that carry a risk of bleeding as a side effect. If a patient with abdominal pain is taking such medications, the possibility of an intestinal wall hematoma should also be considered as a potential cause of symptoms.

The clinical symptoms of colonic hematoma vary and are non-specific, ranging from mild crampy abdominal pain to hemorrhagic shock. Nausea and vomiting, which are detected in 50% of the cases, are related to intestinal obstruction [10]. CECT combined with colonoscopy is the preferred diagnostic method for identifying colonic intramural hematomas. On CT, a hematoma can be identified by a low-absorption area on the periphery, a high-absorption area in the interior, well-defined margins, round or oval shape, and no contrast effect in the absence of active bleeding [11,12]. Submucosal tumors can be mistaken for intestinal intramural hematomas. However, in the case of submucosal tumors, a small amount of contrast enhancement can be observed on CECT. CECT also helps to evaluate active bleeding and determine whether emergency surgery is necessary [12]. However, non-contrast CT should be performed in addition to CECT because bowel wall hemorrhage may be obscured by contrast [13]. Colonoscopy is a valuable diagnostic modality that shows localized intramural hematomas as round, dark reddish submucosal masses or lesions that often cause luminal obstruction [10]. When the patient is stable, CECT and colonoscopy can help in understanding the diagnosis and pathophysiology of this disease. 

The treatment of intestinal intramural hematoma remains controversial. The primary benefit of conservative treatment for intestinal intramural hematomas is the avoidance of surgery-related risks, particularly in older adults or those with underlying diseases that increase the risk of bleeding. In cases of intestinal intramural hematoma caused by anticoagulant therapy, conservative treatment, which includes discontinuing anticoagulants and using antagonists such as vitamin K and warfarin, proved effective in curing one-third of cases [12]. The PT-INR is often prolonged during warfarin-associated bleeding events; therefore, monitoring this with a blood examination can help determine whether conservative treatment is effective. However, no coagulation tests reflect the effects of DOACs, making it difficult to assess the risk of bleeding associated with conservative treatment. Recently, neutralizers for DOACs have been developed and demonstrated to effectively control bleeding tendencies [14]. However, they are only used in cases of life-threatening bleeding, and their effectiveness in cases where conservative treatment is considered is doubtful. Moreover, conservative treatment typically requires several weeks of anticoagulant interruption [15], which increases the associated risk of thrombosis.

Surgical treatment is advantageous because it shortens hospitalization and allows for a definitive diagnosis. Intestinal hematomas can be caused by submucosal tumors or malignant disease, so surgery is also beneficial when a preoperative diagnosis of intramural hematoma is difficult [16]. Bleeding from the hematoma can persist during conservative treatment [17], and intestinal wall hematoma can be complicated by intestinal ischemia or bowel perforation [1,17]. Therefore, if a hematoma increases during conservative treatment or is accompanied by peritonitis, prompt surgical intervention should be considered. However, given that coagulation therapy raises the risk of uncontrolled bleeding during surgery, it is essential to select a treatment method that comprehensively weighs the risks and benefits based on clinical symptoms, blood tests, and imaging findings. In this case, we suspected a hematoma in the intestinal wall based on imaging findings. However, the lesion did not shrink with conservative treatment and the possibility of concurrent malignant disease could not be ruled out, we believed that surgery should be performed.

Laparoscopic colectomy is a surgical procedure that has been developed and is becoming increasingly popular. Several randomized controlled trials have shown that minimally invasive surgery results in better outcomes than open colorectal surgery in terms of reduced blood loss during surgery, less post-operative pain, and shorter hospital stays [18]. Laparoscopic surgery for intestinal obstruction can be challenging because of the limited space in the abdominal cavity. However, if the intestine is sufficiently decompressed by a long tube, the surgery can be completed without much difficulty. However, if intraoperative bleeding is difficult to control, laparotomy should be performed immediately to ensure safe hemostasis. If emergency surgery is necessary because of unstable vital signs or peritonitis, laparotomy should be performed at the beginning to reduce the overall operation time.

While the risk of bleeding from oral DOACs is low, complications such as those observed in this case are expected to become more frequent in the future owing to the increasing number of DOAC prescriptions. Currently, there is no established treatment for intestinal wall hematomas during DOAC administration. It is crucial to thoroughly discuss the treatment strategy, including whether to adopt a conservative or surgical approach, as well as deciding between open or laparoscopic surgery, with the team to select the appropriate treatment method.

## Conclusions

In this study, we successfully performed a laparoscopic right hemicolectomy for an ascending colon intramural hematoma caused by DOACs, and the patient had a good outcome. Although this is a relatively rare complication of DOAC use, it is likely to become more common in the future and should be considered in the differential diagnosis of acute abdomen during anticoagulant therapy. As no standard treatment has been established, selecting an appropriate treatment is vital after a thorough assessment of the patient's condition.
